# Noncystic fibrosis bronchiectasis: Is it an orphan disease?

**DOI:** 10.4103/1817-1737.30353

**Published:** 2007

**Authors:** Khalid F. Mobaireek

**Affiliations:** College of Medicine, King Saud University, Saudi Arabia

Bronchiectasis is a chronic lung disease, defined pathologically as irreversible dilatation of the bronchi; it was first described by Laennec in 1819.[1,2] Orphan disease is a disease that is so rare that it is not considered commercially viable to develop drugs to treat it.[3] Disease is considered orphan either because it is rare or it is more prevalent in developing countries than in the developed world. Bronchiectasis unrelated to cystic fibrosis (CF) was termed an ‘orphan disease.’[4] The hospital admissions for bronchiectasis declined in the developed countries since the 1950s. This decline has been attributed to improved sanitation, nutrition, childhood immunization, and the early and frequent use of antibiotics.[5] However, over the last decade more children are being diagnosed as non-CF bronchiectasis due to the increased use of high resolution computerized tomography.[5] There is a significant under-diagnosis because of low clinical suspicion. The current prevalence in the developed countries is estimated to be 172–335/million children.[5] Though infections remained as an important cause, the causative organisms have changed from tuberculosis, measles and pertussis in the past to adenovirus, herpes and HIV in the present time. Adam[6] reviewed cases of recurrent or persistent pneumonia without apparent cause in all children admitted to King Khalid University Hospital in Riyadh from 1986 to 1989. Out of 781 patients admitted with pneumonia, 18 children met the study inclusion criteria. Children known to have hematological diseases, malignancy, nutritional disorders and immune disorders were excluded. All had associated etiologies: 33% had immunodeficiency, 28% had primary structural and functional anomalies and tumors of the respiratory tract and great vessels, 22% had infections, 11% had idiopathic pulmonary fibrosis and 6% had asthma. In this issue, Dr. Banjar[7] reviewed the experience at King Faisal Specialist Hospital and Research Centre from 1993 to 2005. She found associated disease in 91 (60%) of the cases. These associated diseases included pulmonary diseases in 48 (32%), immunodeficiency in 27 (18%), CNS anomalies in 10 (7%) and cardiac abnormalities in 10 (7%). These are not different from the causes in the developed countries. Measles, pertussis and tuberculosis are no longer a major cause of bronchiectasis in Saudi Arabia as immunization is almost universal and nutrition has improved. The availability of more sophisticated, less invasive and more sensitive radiological studies for bronchiectasis will lead to increased detection of the disease throughout the world. Earlier diagnosis of even mild disease will also change the outcomes, including the possibility of complete resolution. The definition of bronchiectasis may also need revision as the disease may not be permanent in all cases. With these changes, research needs to be encouraged and this frustrating disease should not be orphan any more.[8,9] For this the *Annals of Thoracic Medicine* published a review article[1] in its first issue; and in this issue, an original study[7] besides this editorial – all about bronchiectasis.
